# Incidence, clinical characteristics, risk factors and outcomes of patients with mixed *Candida*/bacterial bloodstream infections: a retrospective study

**DOI:** 10.1186/s12941-022-00538-y

**Published:** 2022-11-01

**Authors:** Li Zhong, Zhaohui Dong, Fengqi Liu, Haidong Li, Kankai Tang, Cheng Zheng, Lifang Wang, Kai Zhang, Jiachang Cai, Hongwei Zhou, Wei Cui, Yanqiu Gao, Gensheng Zhang

**Affiliations:** 1 Department of Critical Care Medicine, First Affiliated Hospital, Huzhou Teachers College, The First People’s Hospital of Huzhou, Huzhou, 313000 Zhejiang China; 2 Department of Critical Care Medicine, Second Affiliated Hospital, Zhejiang University School of Medicine, Hangzhou, 310009 Zhejiang China; 3 Department of Spine Surgery, First Affiliated Hospital, Huzhou Teachers College, The First People’s Hospital of Huzhou, Huzhou, 313000 China; 4 Department of Critical Care Medicine, Taizhou Municipal Hospital, Taizhou, 318000 Zhejiang China; 5 Department of General Medicine, Jiading District Central Hospital Affiliated Shanghai University of Medicine & Health Science, Shanghai, 201800 China; 6 Clinical Microbiology Laboratory, Second Affiliated Hospital, Zhejiang University School of Medicine, Hangzhou, 310009 China; 7 Respiratory Intensive Care Unit, Zhengzhou Central Hospital Affiliated to Zhengzhou University, Zhengzhou, 450007 China; 8 Zhejiang Province Clinical Research Center for Emergency and Critical Care Medicine, Hangzhou, 310009 China

**Keywords:** Candidemia, Bloodstream infections, Mixed *Candida*/bacterial bloodstream infections, *Candida albicans*, Mortality, Risk factor

## Abstract

**Purpose:**

The mixed *Candida*/bacterial bloodstream infections (mixed C/B-BSIs) is worthy of particular attention recently, and we analyzed the incidence, co-pathogens, clinical characteristics, risk factors, and outcomes of mixed C/B-BSIs compared with monomicrobial candidemia (mono-candidemia) in adult patients in China.

**Methods:**

All hospitalized adults with candidemia were recruited for this retrospective observational study from January 1, 2013, to December 31, 2019.

**Results:**

Of the 296 patients with candidemia, 78 cases (26.3%) were mixed C/B-BSIs. *Candida albicans* (*C. albicans*) was the most common *Candida* species among all candidemia, and *Klebsiella pneumoniae* (*K. pneumoniae*) was the most concomitant bacteria (30.6%), followed by *Acinetobacter baumannii* (*A. baumannii*) (12.9%) and *Enterococcus faecium* (*E. faecium*) (11.8%) in mixed C/B-BSIs. In the multivariable analysis, prior β-lactams exposure [adjusted odds ratio (aOR), 1.97; 95% confidence interval (CI), 1.01–3.87], burn injury (aOR, 6.35; 95% CI 1.82–22.21) and continuous renal replacement therapy (CRRT) (aOR, 3.00; 95% CI 1.46–6.17) were independent risk factors for mixed C/B-BSIs. Compared with mono-candidemia, patients with mixed C/B-BSIs developed with more proportion of septic shock (55.1% vs. 39.9%, *P* < 0.05), prolonged stay in ICU [22.0(12.0–57.0) vs. 9.5(0.0–37.0) days, *P* < 0.001] and longer mechanical ventilation time [19.0(4.5–40.8) vs. 6.0(0.0–24.8) days, *P* < 0.001]. The in-hospital mortality in patients with mixed C/B-BSIs was higher than those with mono-candidemia (59.0% vs. 34.9%, *P* < 0.001). Survival analysis revealed that 28-day and 60-day mortality were significantly higher in patients with mixed C/B-BSI than in those with mono-candidemia (57.7% vs. 31.7%, *P* < 0.001; 59.0% vs. 34.9%, *P* < 0.001; respectively).

**Conclusions:**

There is a high rate of mixed C/B-BSIs cases among candidemia, and *K. pneumoniae* is the predominant coexisting species. Prior β-lactams exposure, burn injury, and CRRT are independent risk factors for mixed C/B-BSIs. The mortality of patients with mixed C/B-BSIs is significantly higher than those with mono-candidemia, this deserves further attention for clinicians.

**Supplementary Information:**

The online version contains supplementary material available at 10.1186/s12941-022-00538-y.

## Introduction

Candidemia is associated with a high mortality rate ranging from 19 to 38%, which is one of the most common healthcare-associated bloodstream infections (HA-BSIs) in European and United States surveillance studies [1, 2]. The length of hospital stay increased from 3 to 30 days, and the medical expenses increased from $6214 to $142,394 per patient attributed to *Candida* infections from 1998 to 2000, which exceeded that of the level of most HA-BSIs [2]. Thus, candidemia is a common and serious disease, which puts a great threat to public health.

With the widespread use of broad-spectrum antibiotics, prolonged length of stay, prolonged length of ICU stay, presence of central venous catheter (CVC), and other invasive procedures, the mixed *Candida*/bacterial bloodstream infections (mixed C/B-BSIs) have become prevalent [3, 4]. Blood infections associated with polymicrobial fungal/bacterial represent a huge challenge due to the intrinsic heterogeneity of these consortia, the low susceptibility to traditional drugs, as well as the high toxicity of many common antifungals [5]. Survival rate is similar regardless of concurrent bacteremia, although mixed C/B-BSIs show a lower clearance rate of candidemia during the early period of antifungal therapy, whereas others have reported conflicting results [4]. Recently, Chen et al. [4] have reported that previous hospital stay ≥ 28 days, organic damage during candidemia, and positive procalcitonin (PCT) test were risk factors of mixed C/B-BSIs, and showed concomitant bacteremia was a predictive factor of 30-day mortality of candidemia. However, this study simply focused on candidemia in patients with hematology diseases rather than for all candidemia patients. In addition, the samples of this previous literature regarding such polymicrobial BSIs are relatively small, and whether the concomitant bacteria have some effect on the *Candida* susceptibility in mixed C/B-BSIs and whether there are some differences in the distribution proportion of *Candida* species between the two groups was not mentioned. Thus, the clinical features and outcomes of mixed C/B-BSIs are still largely unknown and need more study to investigate.

To further address these issues, we conducted this retrospective study among the patients with mixed C/B-BSIs and patients with mono-candidemia. As far as I know, this is the largest sample size of mixed C/B-BSIs in China.

## Material and methods

### Patients and study design

A retrospective analysis on candidemia episodes in adult inpatients (≥ 18 years) was conducted in a teaching hospital with 3200 beds between January 1, 2013, and December 31, 2019, and the data were collected from the microbiology database. The Human Ethics Board of the Ethics Committee of the Second Affiliated Hospital of Zhejiang University Medical College authorized the study (No. 2019–191), and written informed consent was not required because of the observational nature of this study.

### Data collection

All data were collected from electronic medical records and were analyzed between groups of patients with mono-candidemia and patients with mixed C/B-BSIs. Demographic data including age, gender, underlying diseases, comorbidities, the severity of illness in the first 24 h following candidemia onset were collected. Data on the life-sustaining treatments ≥ 24 h, prior use of antibiotics or antifungal agents, previous treatments such as surgical procedures, source control were collected. Furthermore, the biological indicators including blood routine test, liver function, serum creatinine, procalcitonin, and C-reactive protein (CRP) at the onset of candidemia were recorded. Microbiological data, such as co-pathogens in mixed C/B-BSIs, the distribution of the *Candida* species, the source of candidemia, and the in-vitro antifungal susceptibility to *Candida* were also recorded. Additionally, outcomes like 28-day, 60-day, and in-hospital mortality were also evaluated.

### Species identification and antimycotic sensitivity test

Species identification of both bacteria and yeasts were performed by matrix-assisted laser desorption/ionization-time of flight mass spectrometry (MALDI-TOF MS) (Bruker Daltonik GmbH, Bremen, Germany). Antimicrobial susceptibility testing for bacteria was carried out with a Vitek 2 Compact system. Susceptibility testing for flucytosine, amphotericin B, fluconazole, voriconazole, and itraconazole was performed using the ATB^®^ FUNGUS 3 system (BioMérieux, France). The susceptibility to antifungal agents and antibiotic agents were provided according to breakpoints defined by the Clinical Laboratory Standards Institute [10, 11]. Because echinocandins were not included in the ATB FUNGUS 3 panel, the results of caspofungin susceptibility were unknown.

### Definitions

Candidemia was defined as the isolation of *Candida* spp. in at least one blood culture in a patient with temporally related clinical signs [12, 13]. Mixed C/B-BSIs was defined as the isolation of a bacterial organism from blood cultures obtained within 48 h before or after the onset of candidemia [3]. When common skin flora (e.g., *coagulase-negative* staphylococci (CoNS), *Bacillus* spp., *Corynebacterium* spp.) appears in at least two separate blood draws or from two separate sites on the same or two consecutive calendar days, and the patient has at least one of the following signs or symptoms: fever (> 38.0 °C), chills, or hypotension when collecting specimens, which can be considered as pathogenic microorganisms [14, 15]. Indirect evidence of fungal infections included the presence of the serological biomarkers 1,3-β-D-glucan (BDG). BDG was defined as positive with a cutoff value of 80 pg/ml [16]. Catheter-related bloodstream infection (CRBSI) was defined according to the Clinical Practice Guidelines for the Diagnosis and Management of Intravascular Catheter-Related Infection [17]. Primary BSI refers to candidemia for which no source of infection can be assigned [14]. The timing of antifungal administration was defined as the interval between the time at which the first *Candia*-positive blood sample for culture was drawn and the time at which antifungal treatment was initially administered [18]. Appropriate antifungal therapy was considered if the isolated *Candida* spp. was sensitive to the chosen antifungal agent and the antifungal agent was administered with an adequate dosage (for example, fluconazole was administered with a loading dose of 800 mg [12 mg/kg] followed by 400 mg [6 mg/kg] daily, or caspofungin was administered with a loading dose of 70 mg followed by 50 mg daily) [19–21]. Meanwhile, the antifungal agent is applicable to the source of the infection site (for example, patients with candidemia and suspected to be of endocardial or central nervous system (CNS) origin should receive Amphotericin B (AmB) (for endocardial or CNS candidiasis) or an echinocandin (for endocardial candidiasis) rather than fluconazole as initial therapy) [20, 21]. A delay in empiric antifungal treatment was considered when initial administration occurred more than 12 h after the first positive blood sample was drawn [18]. Appropriate antimicrobial therapy was defined as the administration of at least one antimicrobial agent to which the causative pathogen was susceptible in vitro within 48 h after the onset of bacteremia and applicable to the source of the infection site, with an approved route and dosage appropriate for end organ(s) function [22]. Prior antimicrobial therapy referred to any antibiotic used for more than 48 h in the past 30 days before candidemia [23]. Immunosuppression conditions include chemotherapy or radiotherapy within 30 days prior to culture, solid organ transplantation or hematopoietic stem cell transplantation within 30 days prior to culture, and corticosteroid therapy with prednisone equivalent at a daily dose of ≥ 25 mg for more than 1 month or a cumulative dose of > 700 mg within 3 months before candidemia onset [24]. Septic shock was consistent with the Third International Consensus Definitions for Sepsis and Septic Shock (Sepsis-3) [25].

### Statistical analyses

SPSS 20.0 software (IBM Corp, Armonk, NY, USA) was performed for statistical analysis. Normal quantitative variables are presented as mean ± standard deviation, while non-normally distributed variables are presented as median and interquartile ranges (IQR). Quantitative variables were compared using the Student *t* test or the Mann–Whitney *U* test, and categorical variables were compared using the Pearson *χ*^2^ or Fisher exact test, where appropriate. To identify the independent risks factors for mixed C/B-BSIs, a multivariate analysis was conducted using a logistic regression model that included variables with *P* < 0.05 in the univariate analysis. The 28-day survival curves of mono-candidemia and mixed C/B-BSIs were depicted by a Kaplan–Meier survival analysis, and the difference was evaluated by the log-rank test. A two-tailed *P* < 0.05 was considered statistically significant.

## Results

**Fig. 1:**
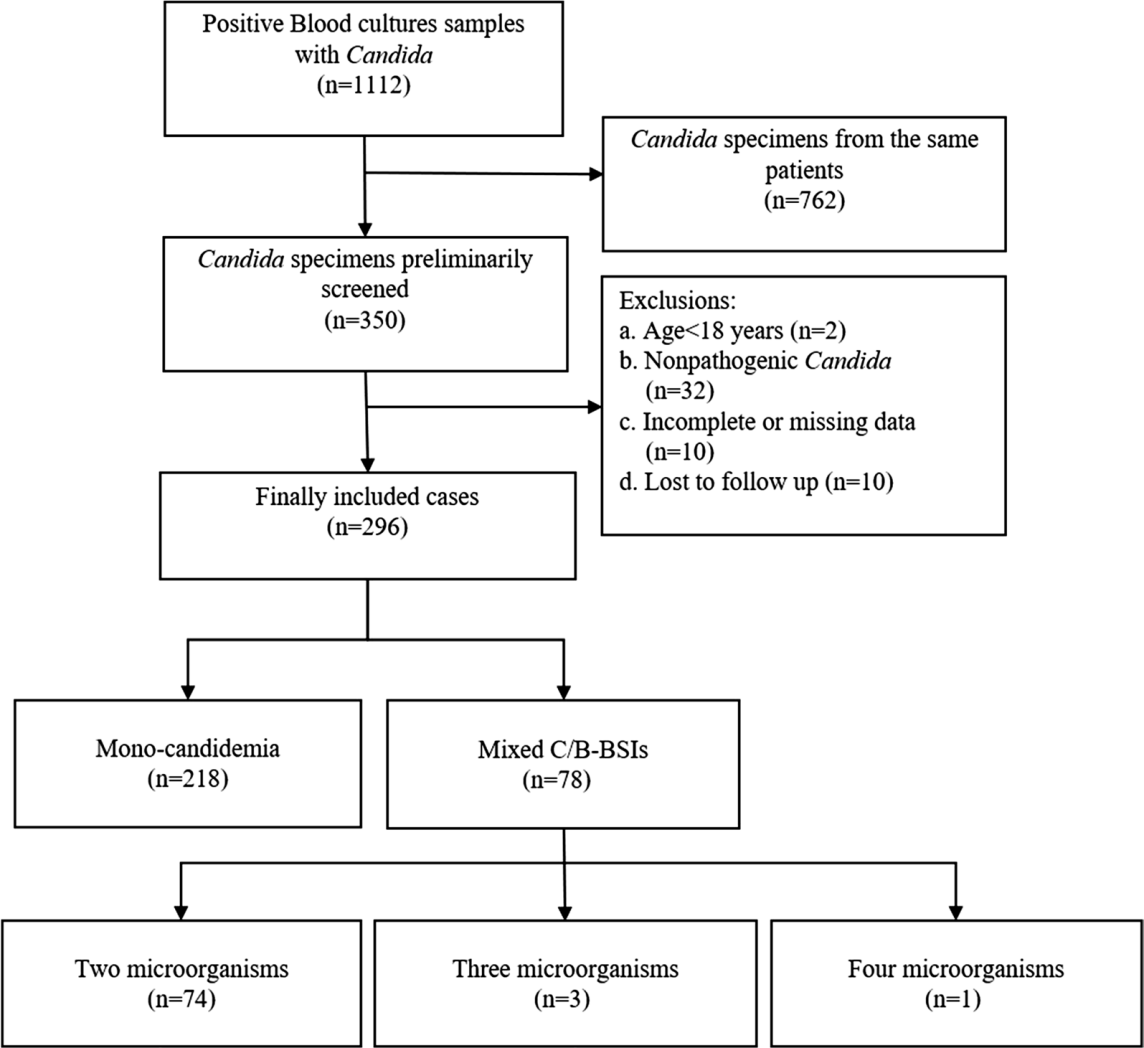
Flowchart of study participant enrollment. Mono-candidemia, monomicrobial candidemia; Mixed C/B-BSIs, mixed *Candida*/bacterial bloodstream infections

A total of 1112 positive *Candida* blood culture specimens were initially analyzed by reviewing the microbiological laboratory data between January 2013 and December 2019. If *Candida* was found in multi*p*le blood cultures of the same patient, only data when the patient first developed candidemia was recruited. After excluding 762 repeated *Candida* cases, a total of 350 positive cases were initially screened. Exclusion criteria were as follows: (a) age < 18 years old; (b) non-pathogenic *Candida*; (c) the case data were incomplete or missing; (d) lost to follow up. Consequently, 54 cases were excluded, including 2 patients less than 18 years old, 32 patients with non-pathogenic *Candida*, 10 patients with incomplete or missing data, 10 patients were lost to follow up. Finally, a total of 296 cases were recruited, with 78 cases of mixed C/B-BSIs and 218 cases of mono-candidemia, respectively (Fig. 1).

### Demographics and clinical characteristics

A significantly higher percentage of burn injury, a longer length of ICU stay or hospital stay before candidemia, a more severe condition and higher rate of life-sustaining treatments was observed in patients with mixed C/B-BSIs than patients with mono-candidemia (all *P* < 0.05) (Table 1).

**Table 1:** Baseline characteristics of the patients with mono-candidemia and mixed C/B-BSIs

Characteristics	Total (n = 296)	mono-candidemia (n = 218)	mixed C/B-BSIs (n = 78)	*P* value
Age, median years (IQR)	65.0 (53.0–75.0)	67.0 (53.0–75.0)	63.5 (51.0–75.5)	0.45
Male sex [n (%)]	188 (63.5)	132 (60.6)	56 (71.8)	0.08
APACHE II score at the onset of candidemia (mean ± S.D.)	21.9 ± 7.4	21.8 ± 7.2	22.2 ± 7.8	0.71
SOFA score at the onset of candidemia (IQR)	6.0 (3.0–9.0)	5.0 (3.0–8.0)	7.0 (4.0–10.3)	** < 0.01**
GCS score at the onset of candidemia (IQR)	15.0 (8.0–15.0)	15.0 (8.0–15.0)	13.0 (6.8–15.0)	**0.02**
Prior ICU stay (days) (IQR)	5.0 (0.0–19.0)	2.0 (0.0–17.0)	11.0 (1.0–22.3)	** < 0.01**
Prior hospital stay (days) (IQR)	15.5 (6.0–31.8)	14.0 (5.0–30.3)	19.0 (10.0–35.5)	**0.01**
Underlying disease [n (%)]				
Diabetes mellitus	51 (17.2)	39 (17.9)	12 (15.4)	0.61
Chronic cardiac dysfunction	51 (17.2)	32 (14.7)	19 (24.2)	0.05
Chronic obstructive pulmonary disease	18 (6.1)	13 (6.0)	5 (6.4)	0.89
Chronic renal insufficiency	27 (9.1)	22 (10.1)	5 (6.4)	0.33
Chronic hepatic insufficiency	29 (9.8)	24 (11.0)	5 (6.4)	0.24
Solid tumour	77 (26.0)	61 (28.0)	16 (20.5)	0.20
Haematological malignancy	11 (3.7)	9 (4.1)	2 (2.6)	0.78
Trauma	51 (17.2)	33 (15.1)	18 (23.1)	0.11
Burn injury	15 (5.1)	7 (3.2)	8 (10.3)	**0.03**
Transplant	4 (1.4)	2 (0.9)	2 (2.6)	0.61
Cerebrovascular accident	99 (33.4)	74 (33.9)	25 (32.1)	0.76
Immunocompromised [n (%)]				
Immunosuppressant therapy	9 (3.0)	7 (3.2)	2 (2.6)	> 0.99
Steroid therapy	16 (5.4)	13 (6.0)	3 (3.8)	0.68
Chemotherapy/radiation	44 (14.9)	35 (16.1)	9 (11.5)	0.34
Neutropenia	12 (4.1)	9 (4.2)	3 (3.9)	0.91
Hospitalization ward [n (%)]				
Medical	44 (14.9)	36 (16.5)	8 (10.3)	0.18
Surgical	60 (20.3)	49 (22.5)	11 (14.1)	0.11
ICU	191 (64.5)	133 (61.0)	58 (74.4)	**0.03**
Life-sustaining treatments ≥ 24 h [n (%)]				
Invasive mechanical ventilation	179 (60.5)	121 (55.5)	58 (74.4)	** < 0.01**
Vasopressor	113 (38.4)	76 (35.0)	37 (48.1)	**0.04**
CRRT	74 (25)	42 (19.3)	32 (41.0)	** < 0.001**
ECMO	5 (1.7)	1 (0.5)	4 (5.3)	**0.02**
Catheterisation ^a^[n (%)]				
Central venous catheter^b^	240 (81.1)	172 (78.9)	68 (87.2)	0.11
Hemodialysis catheter^c^	36 (12.2)	22 (10.1)	14 (17.9)	0.07
PICC	54 (18.2)	39 (17.9)	15 (19.2)	0.79
Peripheral arterial catheters	93 (31.4)	61 (28.0)	32 (41.0)	**0.03**
Drainage tube				
Abdominal drainage tube	83 (28.0)	64 (29.4)	19 (24.4)	0.40
Biliary drainage tube	10 (3.4)	7 (3.2)	3 (3.8)	> 0.99
Thoracic drainage tube	47 (15.9)	29 (13.3)	18 (23.1)	**0.04**
Intracranial drainage tube	47 (15.9)	34 (15.6)	13 (16.7)	0.82
Urethral catheter	263 (88.9)	191 (87.6)	72 (92.3)	0.26
Presence of two or more catheters	260 (87.8)	186 (85.3)	74 (94.9)	**0.03**
Presence of three or more catheters	195 (65.9)	135 (61.9)	60 (76.9)	**0.02**
Presence of two or more central venous catheters	60 (20.3)	38 (17.4)	22 (28.2)	**0.04**
Total parenteral nutrition [n (%)]	198 (66.9)	137 (62.8)	61 (78.2)	**0.01**
Blood transfusion [n (%)]	105 (35.5)	67 (30.7)	38 (48.7)	** < 0.01**
Surgery [n (%)]	164 (55.4)	114 (52.3)	50 (64.1)	0.07
Abdominal	63 (21.3)	46 (21.1)	17 (21.8)	0.90

Bold, indicates *P* < 0.05

*IQR* interquartile range; *COPD* chronic obstructive pulmonary disorder; *SOFA* Sequential Organ Failure Assessment; *GCS* Glasgow coma scale; *APACHE* Acute Physiology and Chronic Health Evaluation; *ICU* intensive care unit; *CRRT* continuous renal replacement therapy; *ECMO* extracorporeal membrane oxygenation; *PICC* peripherally inserted central catheters

^a^Included patients who were required to be catheterised within 2 weeks of the first positive sample, regardless of whether or not the catheter was removed before diagnosis

^b^Non-tunneled central venous catheters such as subclavian, internal jugular and femoral venous catheters excluding hemodialysis catheter and PICC

^c^Non-tunneled temporary dialysis catheter

### The source of candidemia, prior antibiotic and antifungal therapy of the candidemia

The most frequent source of candidemia in mono-candidemia was CVCs (38.1%, 83/218), while it was the primary source in mixed C/B-BSIs (33.3%, 26/78). There was no significant difference in the sources of *Candida* between these two groups (Table 2). The rate of antibiotic exposure prior to candidemia, initial antifungal use with echinocandin, appropriate antifungal therapy in patients with mixed C/B-BSIs were higher than those with mono-candidemia (all *P* < 0.05). A lower percentage of delay in initiation of empiric antifungal treatment was observed in patients with mixed C/B-BSIs than mono-candidemia (*P* < 0.05). There were no significant differences in the rate of infection source control and prior antifungal exposure between the two groups (Table 2).

**Table 2:** The source of candidemia, prior antibiotic and antifungal therapy of the mixed C/B-BSIs compared with the mono-candidemia

Variable	Total (n = 296)	Mono-candidemia (n = 218)	Mixed C/B-BSIs (n = 78)	*P* value
Source of candidemia [n (%)]				
Definitive CVC-related	108 (36.5)	83 (38.1)	25 (32.1)	0.34
Primary	79 (26.7)	53 (24.3)	26 (33.3)	0.12
Intra-abdominal	43 (14.5)	34 (15.6)	9 (11.5)	0.38
Lower respiratory tract	22 (7.4)	15 (6.9)	7 (9.0)	0.54
Gastrointestinal tract	18 (6.1)	12 (5.5)	6 (7.7)	0.68
Urinary tract infection	15 (5.1)	13 (6.0)	2 (2.6)	0.38
Skin and Soft tissue	8 (2.7)	6 (2.8)	2 (2.6)	> 0.99
Meningitis	2 (0.7)	1 (0.5)	1 (1.3)	0.46
Endocardium	1 (0.3)	1 (0.5)	0 (0.0)	> 0.99
Source control [n (%)]				
Removal of contaminated lines^a^	198 (67.6)	144 (66.7)	54 (70.1)	0.58
Draining of fungal collection	65 (22.0)	46 (21.1)	19 (24.4)	0.55
Prior antibiotic exposure ^b^ [n (%)]	235 (79.4)	164 (75.2)	71 (91.0)	** < 0.01**
Cephalosporins	81 (27.4)	54 (24.8)	27 (34.6)	0.10
Carbapenems	113 (38.2)	86 (39.4)	27 (34.6)	0.45
Penicillins	60 (20.3)	41 (18.8)	19 (24.4)	0.30
β-lactams	102 (34.5)	66 (30.3)	36 (46.2)	**0.01**
Quinolones	14 (4.7)	11 (5.0)	3 (3.8)	0.91
Initial antifungal agent [n (%)]				
Fluconazole	112 (37.8)	85 (39.0)	27 (34.6)	0.49
Echinocandin	112 (37.8)	74 (33.9)	38 (48.7)	**0.02**
Voriconazole	35 (11.8)	30 (13.8)	5 (6.4)	0.08
Prior antifungal exposure [n (%)]	35 (11.8)	22 (10.1)	13 (16.7)	0.12
Prior azole antifungal exposure [n (%)]	21 (7.1)	12 (5.5)	9 (11.5)	0.07
Appropriate antifungal therapy ^c^ [n (%)]	39 (13.3)	21 (9.7)	18 (23.4)	** < 0.01**
Delay in initiation of empiric antifungal treatment ^d^ [n (%)]	203 (68.6)	158 (72.5)	45 (57.7)	**0.02**

*CVC* central venous catheter; *PICC* peripherally inserted central catheters; *CRBSI* catheter-related bloodstream infection; *PCT* procalcitonin test

Bold, indicates *P* < 0.05

^a^Central venous catheter removed within 48 h after the first positive sample

^b^All patients receiving systemic drug therapy for ≥ 3 days within 2 weeks prior to candidaemia onset

^c^Antifungal therapy was defined as appropriate if the isolated *Candida* spp was sensitive to the chosen antifungal agent, and the antifungal agent was used with adequate dosages (like Fluconazole: loading dose of 800 mg [12 mg/kg], then 400 mg [6 mg/kg] daily; Caspofungin: loading dose of 70 mg, then 50 mg daily)

^d^The delay of empiric antifungal treatment was considered as initial use more than 12 h after the report of first positive blood sample

### Biological indicators

In comparison with mono-candidemia, patients with mixed C/B-BSIs had a higher glutamic-oxaloacetic transaminase (GOT), higher lactic acid and elevated inflammatory markers evidenced by significant increases in levels of procalcitonin and CRP (all *P* < 0.05) (Table 3).

**Table 3:** Comparison of biological indicators between groups of mono-candidemia and mixed C/B-BSIs

Biological indicators	Total (n = 296)	Mono-candidemia (n = 218)	Mixed C/B-BSIs (n = 78)	*P* value
Procalcitonin (ng/L) (IQR)	0.7 (0.3–2.3)	0.6 (0.2–1.6)	1.2 (0.4–7.0)	** < 0.01**
CRP (mg/L) (IQR)	82.8 (46.6–151.6)	78.5 (42.0–136.8)	90.0 (58.4–171.8)	**0.04**
1,3-β-D glucan test [n (%)]	96 (32.4)			
Positive	34 (11.5)	24 (11.0)	10 (12.8)	0.67
Negative	62 (20.9)	39 (17.9)	23 (29.5)	**0.03**
Liver and kidney function				
GPT (U/L) (IQR)	32.0 (20.0–63.5)	32.0 (21.0–59.3)	36.0 (16.8–82.8)	0.66
GOT (U/L) (IQR)	39.0 (25.0–64.5)	36.0 (24.8–57.0)	44.0 (27.8–99.0)	**0.02**
Albumin (g/L) (mean ± S.D.)	30.7 ± 5.8	31.0 ± 5.8	29.8 ± 5.8	0.15
TBil (μmol/L) (IQR)	16.0 (11.0–30.5)	15.0 (10.0–29.2)	17.0 (11.0–37.0)	0.06
BUN (mmol/L) (IQR)	8.4 (5.1–14.3)	8.1 (4.9–14.9)	8.8 (5.4–12.7)	0.88
SCr (μmol/L) (IQR)	61.0 (40.0–95.8)	59.0 (39.8–93.3)	67.0 (41.8–101.0)	0.39
Blood routine test				
WBC (× 10^9^/L) (IQR)	9.5 (5.8–14.0)	9.2 (5.6–13.2)	10.0 (6.7–15.0)	0.19
Hemoglobin (g/L) (IQR)	82.0 (71.0–95.0)	83.0 (71.0–99.3)	80.0 (68.0–90.3)	0.06
Hematocrit (%) (IQR)	24.4 (21.0–29.0)	25.0 (21.0–30.0)	24.0 (21.0–27.0)	**0.05**
Platelet (× 10^9^/L) (IQR)	130.0 (70.0–230.5)	134.5 (75.8–229.5)	116.5 (46.0–245.0)	0.28
Lactic acid (mmol/L) (IQR)	1.5 (1.0–2.5)	1.5 (1.0–2.3)	1.8 (1.2–3.2)	**0.02**

^The time point for the biological indicators was on the day of submission of the first positive blood culture sample of *candida*^

^Bold, indicates *p *< 0.05^

^*IQR* interquartile range; *CRP* C−reactive protein; *GPT* glutamic−pyruvic transaminase; *GOT* glutamic−oxaloacetic transaminase; *TBil* total bilirubin; *BUN* blood urea nitrogen; *SCr* serum creatinine; *WBC* white blood count^

### Antifungal susceptibility

As shown in Additional file 2: Table S1, the susceptibility of *C. albicans*, *C. parapsilosis* to fluconazole, amphotericin B and voriconazole were quite high. However, there was no significant difference between the mono-candidemia and mixed C/B-BSIs groups in the in-vitro antifungal susceptibility test. Because the drug sensitivity kit used in our current microbiology laboratory does not include echinocandins, the specific drug sensitivity of *Candida* species to echinocandins was unclear.

### Independent risk factors for mixed C/B-BSIs

As shown in Table 4, the independent risk factors for mixed C/B-BSIs were prior β-lactams exposure [ adjusted odds ratio (aOR), 1.97; 95% confidence interval (CI) 1.01–3.87], burn injury (aOR, 6.35; 95% CI 1.82–22.21) and CRRT use (aOR, 3.00; 95% CI 1.46–6.17).

**Table 4:** Multivariable logistic regression of factors associated with mixed C/B-BSIs

Risk factors	B	S.E	Wald	*P* value	aOR (95% CI)
Prior hospital stay (days)	0.00	0.01	0.02	0.88	1.00 (0.99–1.01)
Prior antibiotic exposure	0.88	0.53	2.76	0.10	2.42 (0.85–6.85)
Prior β-lactams exposure	0.68	0.34	3.93	**0.05**	1.97 (1.01–3.87)
Initial antifungal agent was Echinocandin	0.56	0.33	3.01	0.08	1.76 (0.93–3.33)
Prior ICU stay (days)	0.01	0.01	1.15	0.28	1.01 (0.99–1.02)
Burn injury	1.85	0.64	8.38	** < 0.01**	6.35 (1.82–22.21)
ECMO	1.15	1.20	0.92	0.34	3.16 (0.30–33.31)
CRRT	1.10	0.37	8.88	** < 0.01**	3.00 (1.46–6.17)
Invasive mechanical ventilation	0.24	0.43	0.31	0.58	1.27 (0.54–2.98)
Total parenteral nutrition	0.64	0.36	3.10	0.08	1.89 (0.93–3.85)
Blood transfusion	0.39	0.33	1.45	0.23	1.48 (0.78–2.81)
ICU admission	-0.52	0.43	1.41	0.24	0.60 (0.25–1.40)
Lactic acid	0.06	0.06	0.87	0.35	1.06 (0.94–1.19)
Procalcitonin	0.02	0.01	2.34	0.13	1.02 (0.99–1.04)
SOFA score	0.03	0.05	0.48	0.49	1.03 (0.94–1.14)
Peripheral arterial catheters	-0.22	0.37	0.33	0.56	0.81 (0.39–1.68)
Thoracic drainage tube	0.56	0.40	1.95	0.16	1.75 (0.80–3.85)
Constant	-3.86	0.64	36.68	** < 0.01**	0.02 (–)

Bold, indicates *P* < 0.05

*B* coefficient; *SE* standard error; *Wald* wald test statistic; *OR* odds ratio; *CI* confidence interval; *ICU* intensive care unit; *ECMO* extracorporeal membrane oxygenation; *CRRT* continuous renal replacement therapy; SOFA Sequential Organ Failure Assessment

### Species distributions of concomitant bacteria isolated from the mixed C/B-BSIs

**Fig. 2:**
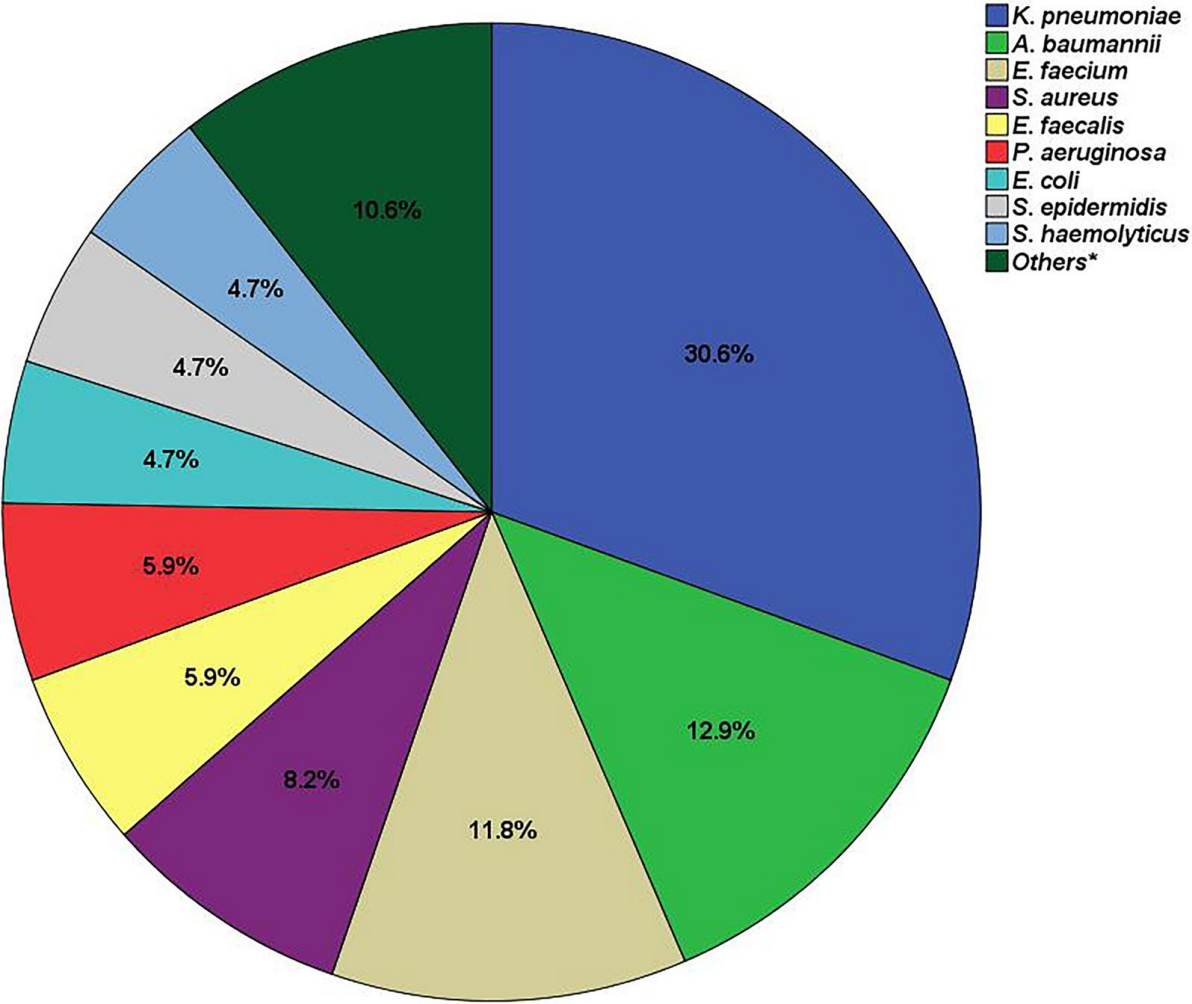
The species distributions of concomitant bacteria isolated from the mixed C/B-BSIs. Mixed C/B-BSIs, mixed *Candida*/bacterial bloodstream infections. *E. aerogenes (n = 1), *S. capitis* (n = 1), *P. mirabilis* (n = 1), *S. maltophilia* (n = 1), *B. cepacia* (n = 1), *P. vulgaris* (n = 1), *K. oxytoca* (n = 1), *E. raffinosus* (n = 1) and *E. avium* (n = 1).

A total of 85 isolates with various bacterial organisms were isolated from the 78 cases (Fig. 2). Among these concomitant bacteria, gram-negative bacteria accounted for 61.2% (52/85), while gram-positive bacteria accounted only for 38.8% (33/85). The most common co-pathogens were *Klebsiella pneumoniae* (*K. pneumoniae*) (30.6%, 26/85), followed by *Acinetobacter baumannii* (*A. baumannii*) (12.9%, 11/85), *Enterococcus faecium* (*E. faecium*) (11.8%, 10/85) and *Staphylococcus aureus* (*S. aureus*) (8.24%, 7/85). The detailed distribution of concomitant bacterial species in mixed C/B-BSIs is shown in Fig. 2.

### The distribution comparison of *Candida* species isolated from mixed C/B-BSIs and mono-candidemia

A total of 296 *Candida* spp. were isolated, and the most common *Candida* species was *C. albicans* (136/296, 45.9%), followed by *C. tropicalis* (69/296, 23.3%), *C. parapsilosis* (47/296, 15.9%), *C. glabrata* (33/296, 11.1%). Thus, non-albicans species were dominant among candidemia (160/296, 54.1%). The distribution comparison of *Candida* species isolated from mixed C/B-BSIs and mono-candidemia is shown in Additional file 1: Fig. S1, which showed the proportion of *Candida* species was similar in the two groups (all *P* > 0.05).

### Trends in the episodes of candidemia and mixed C/B-BSIs during the study period

**Fig. 3:**
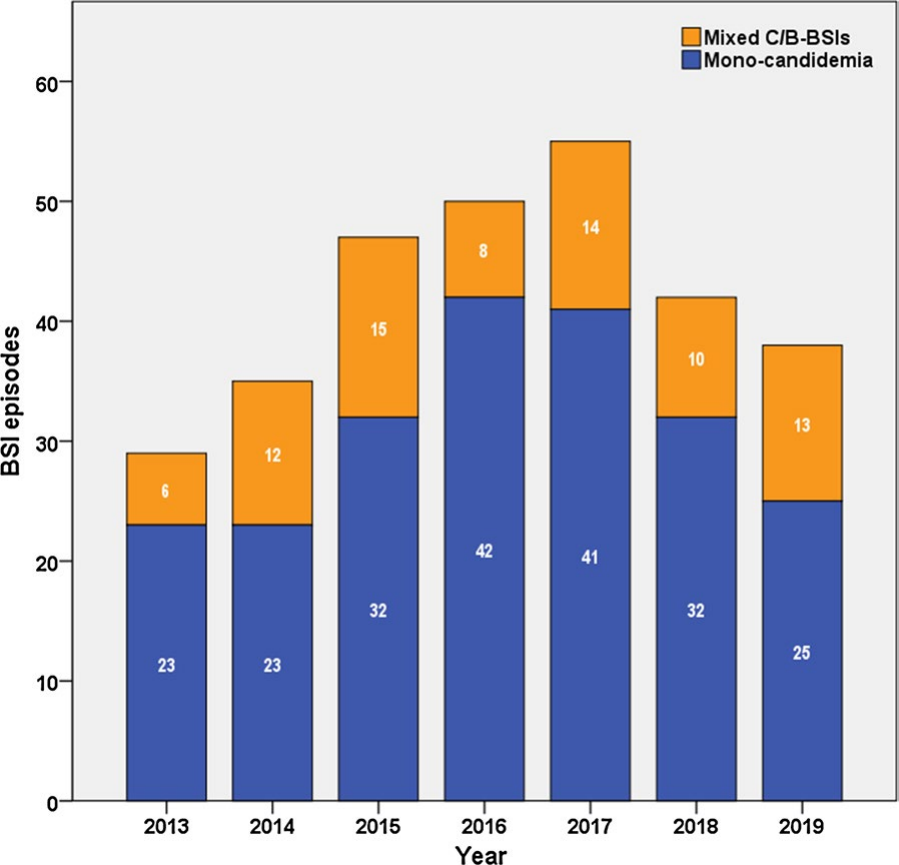
Trends in the episodes of candidemia and mixed C/B-BSIs during the study period. Mixed C/B-BSIs, mixed *Candida*/bacterial bloodstream infections

Next, we analyzed the dynamic changes of incidence of mixed C/B-BSIs during the seven years from 2013 to 2019. The overall incidence of mixed C/B-BSIs showed a relatively stable trend, fluctuating a range of 16.0–34.3% (Fig. 3).

### Outcomes

**Fig. 4:**
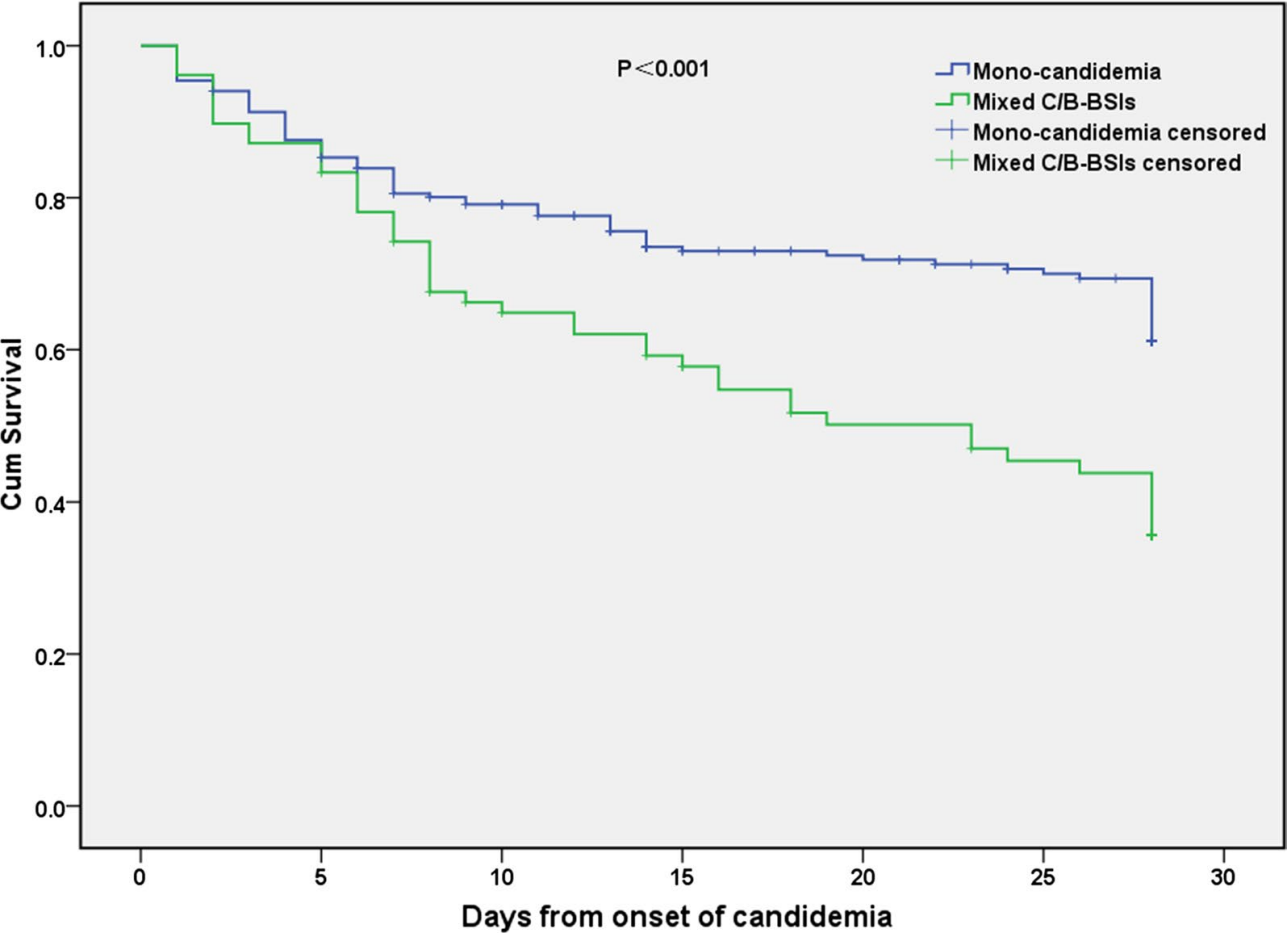
Kaplan–Meier estimates of survival in patients with mixed C/B-BSIs and mono-candidemia. Mono-candidemia, monomicrobial candidemia; Mixed C/B-BSIs, mixed *Candida*/bacterial bloodstream infections

Survival analysis revealed that 28-day and 60-day mortality were significantly higher in patients with mixed C/B-BSI than in those with mono-candidemia (Both *P* < 0.001) (Table5). Furthermore, the Kaplan–Meier curves with the log-rank test revealed a significant difference in 28-day survival after diagnosis of candidemia between patients with mono-candidemia (61.1%) and patients with mixed C/B-BSIs (35.7%, *P* < 0.001) (Fig. 4).

**Table 5:** Comparison of clinical outcomes between mono-candidemia and mixed C/B-BSIs

Outcomes	Total (n = 296)	Mono-candidemia (n = 218)	Mixed C/B-BSIs (n = 78)	*P* value
Total ICU stay days (IQR)	14.0 (1.0–38.0)	9.5 (0.0–37.0)	22.0 (12.0–57.0)	** < 0.001**
Total Hospitalization days (IQR)	36.0 (20.0–66.0)	34.0 (18.8–61.0)	42.5 (22.8–73.5)	0.07
Total mechanical ventilation days (IQR)	10.0 (0.0–32.3)	6.0 (0.0–24.8)	19.0 (4.5–40.8)	** < 0.001**
Septic shock [n (%)]	130 (43.9)	87 (39.9)	43 (55.1)	**0.02**
28-day mortality [n (%)]	104 (35.1)	63 (28.9)	41 (52.6)	** < 0.001**
60-day mortality [n (%)]	114 (38.5)	69 (31.7)	45 (57.7)	** < 0.001**
In-hospital mortality [n (%)]	122 (41.2)	76 (34.9)	46 (59.0)	** < 0.001**

Bold, indicates *P* < 0.05

*ICU* Intensive care unit; *IQR* Interquartile range

## Discussion

Mixed C/B-BSIs occupied a high proportion of 26.3% among candidemia in the current study, which was consistent with previous studies that 18–56% of nosocomial candidemia are polymicrobial [3, 4, 6–9]. In Kim’s report [3], the mixed *Candida*/bacterial bloodstream infections were accounted for 23% (37/126) of candidemia episodes in a teaching hospital in South Korea. A 30.8% (20/65) frequency of mixed C/B-BSIs in patients with hematological diseases was reported in a national key hematologic center in China [4]. These studies suggest that the proportion of polymicrobial candidemia is not rare, which deserves the attention of clinicians.

In the current study, many factors were associated with mixed C/B-BSIs (Tables 1–3). By multinomial regression analysis, burn injury, CRRT, and β-lactams exposure prior to candidemia were found to be independent factors for mixed C/B-BSIs. Consistent with our current study, patients with burn injury were more susceptible to suffer mixed BSIs like polymicrobial BSI, mixed-enterococcal BSI, and polymicrobial *S. aureus* BSI [26–28], which might be associated with alterations in cellular and humoral immune responses, extensive skin barrier disruption, high possibility of gastrointestinal bacterial translocation, prolonged hospitalization, and invasive diagnostic/therapeutic procedures [29]. Therefore, as a common colonizing pathogen of the skin, *Candida* app. are more likely to invade the blood through the damaged skin and cause bloodstream infections together with other pathogens. Hemodialysis represents a significant risk factor for the development of candidemia, and catheter dialysis has been identified as one of the main independent risk factors [30]. CRRT was an independent risk factor of mixed C/B-BSIs in our research, which was consistent with the previous study, showing that CRRT was associated with the onset of secondary bacterial infection [31]. There are several possible explanations for this issue: (i) Immunodeficiency is common in patients with end-stage renal function [32, 33]; (ii) Disruptions of dermal barriers to gain access for dialysis [34]; (iii) Repeated hemodialysis access to the vascular system through a venous catheter, resulting in frequent episodes of bloodstream infection [31]. Correspondingly, CRBSI has become the leading cause of hospital-acquired bloodstream infection in the kidney ICU [35]. In this study, we found prior β-lactams exposure was independently associated with mixed C/B-BSIs. A similar result was observed where *C. albicans* promote *Enterococcus* populations' recovery following cefoperazone treatment [36]. In theory, our result might be explained by the fact that treatment with β-lactam antibiotics significantly enhanced commensal bacteria of the gut microbiota to release a large number of peptidoglycan fragments, which induce the invasive hyphal growth of *C. albicans*, leading to the penetration of tissue barriers and invasion of internal organs [37].

Among the concomitant bacteria isolated from the mixed C/B-BSIs, gram-negative bacteria were remarkably higher than gram-positive bacteria (61.2% vs. 38.8%) in the current study. In terms of specific species, the most common co-pathogen in mixed C/B-BSIs was *K. pneumoniae* (30.6%), followed by *A. baumannii* (12.9%), *E. faecium* (11.8%), and *S. aureus* (8.24%, 7/85). These results were in agreement with Chen’s findings, which showed gram-negative bacteria were the predominantly concomitant species isolated from the mixed C/B-BSIs [4]. Similarly, the *K. pneumoniae* (35%, 7/20) were the most frequent bacteria combined with a *Candida sp.* in patients with hematological diseases [4]. However, our results were contrary to other previous studies which have suggested that gram-positive bacteria were the predominant species [3, 6, 7]. In Klotz’s surveys [7] which involved 372 patients with candidemia, 24% (88/372) had synchronous bacteremia, and the top three most commonly co-isolated bacterial species were *Staphylococcus epidermidis* (*S. epidermidis*), *Enterococcus spp.*, *and S. aureus.* Kim, et al. [3] showed that Gram-positive organisms accounted for more than 68% (30/44) of all bacterial isolates from the mixed C/B-BSIs, and CoNS (23%, 10/44) were the most prevalent bacterial pathogen. This discrepancy could be attributed to the differences in enrolled participants and sources of BSI. In Kim’s study [3], the most common co-morbidity was solid tumor (46%, 17/37), and the CVC (30%, 11/37) and the gastrointestinal tract (30%, 11/37) were the most common sources of bacteremia. Similarly, a high proportion of mixed C/B-BSIs was attributed to a catheter origin in Bouza’s report [6]. Evidence has revealed that gram-positive bacteria such as CoNS and *S. aureus* are the most common pathogenic species of catheter-related infection [17]. In contrast, the most common co-morbidities in the mixed C/B-BSIs was cerebrovascular accident (32.1%, 35/78) and the most common source of bacteremia was intracranial infection (33%, 26/78) in the current study. Indeed, gram-negative bacteria were the main pathogenic microorganisms of intracranial infection as shown in our previous study [38].

In accordance with the present results, previous studies have demonstrated that patients with mixed C/B-BSIs might have worse outcomes than those with mono-candidemia [4]. The worse outcomes of mixed C/B-BSIs in our study were possibly associated with the following factors: (1) The synergistic relationship between *Candida* and bacteria species enhances the viability of *Candida* or bacterial, which might result in difficulties inefficient treatments and pathogen eradication despite adequate antimicrobial and antifungal therapy [39]. In an experimental study, a synergistic effect of *C. albicans* and *S. aureus* infections on mortality in mice was also observed [40]. (2) *K. pneumoniae* is the most common co-pathogens in mixed C/B-BSIs. It is well-known that carbapenem-resistant *K. pneumoniae* could cause a mortality rate of up to 50% [41]. Indeed, a high percentage of carbapenem-resistant *K. pneumoniae* accounted for up to 28.2% (24/85) in the mixed C/B-BSIs group was observed in our study. (3) Patients with mixed C/B-BSIs were more severe in comparison with mono-candidemia, evidenced by a higher sequential organ failure assessment (SOFA) score (7.0 vs. 5.0, *P* < 0.05), a higher proportion of septic shock (55.1% vs. 39.9%, *P* < 0.05). (4) Higher proportions of CRRT and total parenteral nutrition in patients with mixed C/B-BSIs were observed than those with mono-candidemia (41.0% vs. 19.3%, 78.2% vs. 62.8%, respectively, both *P* < 0.05). Indeed, renal failure and total parenteral nutrition were reported to be associated with high mortality [42].

However, there were some limitations in the present study. First, as a result of the retrospective study itself, some data like patient characteristics or co-morbidities were obtained based on medical records rather than an interview or clinical examination at the time of infection, which might lead to some important information or variables could not be obtained accurately. Second, the current study was performed from a single center and the number of patients was relatively small, though it has reviewed the record of candidemia over 7 years in our hospital. In addition, as our institution is well-known in the field of trauma treatment nationwide, there was a considerable number of patients with trauma and burn in the study, which might lead to a selection bias. Third, it is possible that some important confounding variables for mixed C/B-BSIs were not included and analyzed, which is an intrinsic shortcoming for a retrospective study. Thus, multicenter studies with a large sample size are necessary to further investigate the characteristics and risk factors of mixed C/B-BSIs.

## Conclusions

Mixed C/B-BSIs account for a considerable proportion of candidemia. The *K. pneumoniae* is the predominant coexisting species in mixed C/B-BSIs. Prior β-lactams exposure, burn injury, and CRRT are independent risk factors for mixed C/B-BSIs. In addition, patients with mixed C/B-BSIs have worse outcomes compared with mono-candidemia, which might be attracted more attention by physicians in the future.

## Supplementary Information

**Additional file 1: Figure S1.** The distribution comparison of *Candida* species isolated from mixed C/B-BSIs and mono-candidemia. Mono-candidemia, monomicrobial candidemia; Mixed C/B-BSIs, mixed *Candida*/bacterial bloodstream infections.

**Additional file 2: Table S1**. Comparison of vitro antifungal susceptibility of candida between mono-candidemia and mixed C/B-BSIs.

## Data Availability

All data generated or analyzed during this study are included in this manuscript.

## Abbreviations

Mixed C/B-BSIs: Mixed *Candida* /bacterial bloodstream infections
mono-candidemia: Monomicrobial *Candida* bloodstream infection
BSI: Bloodstream infection
IQR: Interquartile range
aOR: Adjusted odds ratio
CI: Confidence interval
CRRT: Continuous renal replacement therapy
CVC: Central venous catheter
COPD: : Chronic obstructive pulmonary disorder
SOFA: Sequential organ failure assessment
APACHE: Acute physiology and chronic health evaluation
ICU: Intensive care unit
OR: Odds ratio
CDC: Centers for disease control and prevention
CLSI: Clinical and laboratory standards institute
BDG: 1,3-β-D-glucan
CoNS: Coagulase-negative staphylococci
CNS: Central nervous system
AmB: Amphotericin B
*K. pneumoniae*: *Klebsiella pneumoniae*
*S. aureus*: *Staphylococcus aureus*
*A. baumannii*: *Acinetobacter baumannii*
*E. faecium*: *Enterococcus faecium*
*P. aeruginosa*: *Pseudomonas aeruginosa*
*B. cepacia*: *Burkholderia cepacia*
*P. mirabilis*: *Proteus mirabilis*
*S. epidermidis*: *Staphylococcus epidermidis*
*E. faecalis*: *Enterococcus faecalis*
*E.coli*: *Escherichiacoli*
*S. maltophilia*: *Stenotrophomonas maltophilia*
*S. haemolyticus*: *Staphylococcus haemolyticus*
*E. aerogenes*: *Enterobacter aerogenes*
*S. capitis*: *Staphylococcus capitis*
*E. raffinosus*: *Enterococcus. raffinosus*
*E. avium*: *Enterococcus avium*
*K. oxytoca*: *Klebsiella oxytoca*
*P. vulgaris*: *Proteus vulgaris*

## References

[1] Koehler P, Stecher M, Cornely OA, Koehler D, Vehreschild M, Bohlius J (2019). Morbidity and mortality of candidaemia in Europe: an epidemiologic meta-analysis. Clin Microbiol Infect.

[2] Morgan J, Meltzer MI, Plikaytis BD, Sofair AN, Huie-White S, Wilcox S (2005). Excess mortality, hospital stay, and cost due to candidemia: a case-control study using data from population-based candidemia surveillance. Infect Control Hosp Epidemiol.

[3] Kim SH, Yoon YK, Kim MJ, Sohn JW (2013). Risk factors for and clinical implications of mixed Candida/bacterial bloodstream infections. Clin Microbiol Infect.

[4] Chen XC, Xu J, Wu DP (2020). Clinical characteristics and implications of mixed candida/bacterial bloodstream infections in patients with hematological diseases. Eur J Clin Microbiol Infect Dis.

[5] Harriott MM, Noverr MC (2011). Importance of Candida-bacterial polymicrobial biofilms in disease. Trends Microbiol.

[6] Bouza E, Burillo A, Munoz P, Guinea J, Marin M, Rodriguez-Creixems M (2013). Mixed bloodstream infections involving bacteria and Candida spp.. J Antimicrob Chemother.

[7] Klotz SA, Chasin BS, Powell B, Gaur NK, Lipke PN (2007). Polymicrobial bloodstream infections involving Candida species: analysis of patients and review of the literature. Diagn Microbiol Infect Dis.

[8] Pulimood S, Ganesan L, Alangaden G, Chandrasekar P (2002). Polymicrobial candidemia. Diagn Microbiol Infect Dis.

[9] Zhong L, Zhang S, Tang K, Zhou F, Zheng C, Zhang K (2020). Clinical characteristics, risk factors and outcomes of mixed *Candida*
*albicans*/bacterial bloodstream infections. BMC Infect Dis.

[10] Institute CaLS. Reference method for broth dilution antifungal susceptibility testing of yeasts, Third informational supplement, M27-A3 Wayne, PA2008.

[11] Institute CaLS. Performance standards for antimicrobial susceptibility testing , 28th ed, supplement M100. Wayne, PA2018.

[12] Charles PE, Dalle F, Aube H, Doise JM, Quenot JP, Aho LS (2005). Candida spp. colonization significance in critically ill medical patients: a prospective study. Intensive Care Med.

[13] Ascioglu S, Rex JH, de Pauw B, Bennett JE, Bille J, Crokaert F (2002). Defining opportunistic invasive fungal infections in immunocompromised patients with cancer and hematopoietic stem cell transplants: an international consensus. Clin Infect Dis.

[14] CDC. Bloodstream infection event (central line-associated bloodstream infection and non-central line-associated bloodstream infection). Atlanta, Georgia: CDC 2015.

[15] CDC. Identifying Healthcare-associated Infections (HAI) for NHSN Surveillance. Atlanta, Georgia: CDC 2015.

[16] Angebault C, Lanternier F, Dalle F, Schrimpf C, Roupie AL, Dupuis A (2016). Prospective evaluation of serum β-glucan testing in patients with probable or proven fungal diseases. Open Forum Infect Dis.

[17] Mermel LA, Allon M, Bouza E, Craven DE, Flynn P, O'Grady NP (2009). Clinical practice guidelines for the diagnosis and management of intravascular catheter-related infection: 2009 update by the infectious diseases society of America. Clin Infect Dis.

[18] Morrell M, Fraser VJ, Kollef MH (2005). Delaying the empiric treatment of candida bloodstream infection until positive blood culture results are obtained: a potential risk factor for hospital mortality. Antimicrob Agents Chemother.

[19] Das I, Nightingale P, Patel M, Jumaa P (2011). Epidemiology, clinical characteristics, and outcome of candidemia: experience in a tertiary referral center in the UK. Int J Infect Dis.

[20] Pappas PG, Kauffman CA, Andes D, Benjamin DK, Calandra TF, Edwards JE (2009). Clinical practice guidelines for the management of candidiasis: 2009 update by the Infectious Diseases Society of America. Clin Infect Dis.

[21] Pappas PG, Kauffman CA, Andes DR, Clancy CJ, Marr KA, Ostrosky-Zeichner L (2016). Clinical practice guideline for the management of candidiasis: 2016 update by the Infectious Diseases Society of America. Clin Infect Dis.

[22] Wang YC, Ku WW, Yang YS, Kao CC, Kang FY, Kuo SC (2020). Is polymicrobial bacteremia an independent risk factor for mortality in *Acinetobacter*
*baumannii* Bacteremia?. J Clin Med..

[23] Lortholary O, Renaudat C, Sitbon K, Desnos-Ollivier M, Bretagne S, Dromer F (2017). The risk and clinical outcome of candidemia depending on underlying malignancy. Intensive Care Med.

[24] Kaech C, Elzi L, Sendi P, Frei R, Laifer G, Bassetti S (2006). Course and outcome of *Staphylococcus*
*aureus* bacteraemia: a retrospective analysis of 308 episodes in a Swiss tertiary-care centre. Clin Microbiol Infect.

[25] Singer M, Deutschman CS, Seymour CW, Shankar-Hari M, Annane D, Bauer M (2016). The third international consensus definitions for sepsis and septic shock (Sepsis-3). JAMA.

[26] Zorgani A, Franka RA, Zaidi MM, Alshweref UM, Elgmati M (2010). Trends in nosocomial bloodstream infections in a burn intensive care unit: an eight-year survey. Ann Burns Fire Disasters.

[27] Zheng C, Zhang S, Chen Q, Zhong L, Huang T, Zhang X (2020). Clinical characteristics and risk factors of polymicrobial *Staphylococcus*
*aureus* bloodstream infections. Antimicrob Resist Infect Control.

[28] Zheng C, Cai J, Liu H, Zhang S, Zhong L, Xuan N (2019). Clinical characteristics and risk factors in mixed-Enterococcal bloodstream infections. Infect Drug Resist.

[29] Church D, Elsayed S, Reid O, Winston B, Lindsay R (2006). Burn wound infections. Clin Microbiol Rev.

[30] Pyrgos V, Ratanavanich K, Donegan N, Veis J, Walsh TJ, Shoham S (2009). Candida bloodstream infections in hemodialysis recipients. Med Mycol.

[31] Powe NR, Jaar B, Furth SL, Hermann J, Briggs W (1999). Septicemia in dialysis patients: incidence, risk factors, and prognosis. Kidney Int.

[32] Vanholder R, Ringoir S (1993). Infectious morbidity and defects of phagocytic function in end-stage renal disease: a review. J Am Soc Nephrol.

[33] Pesanti EL (2001). Immunologic defects and vaccination in patients with chronic renal failure. Infect Dis Clin North Am.

[34] Khan IH, Catto GR (1993). Long-term complications of dialysis: infection. Kidney Int Suppl.

[35] Cheng S, Xu S, Guo J, He Q, Li A, Huang L (2019). Risk factors of central venous catheter-related bloodstream infection for continuous renal replacement therapy in kidney intensive care unit patients. Blood Purif.

[36] Mason KL, Erb Downward JR, Mason KD, Falkowski NR, Eaton KA, Kao JY (2012). *Candida*
*albicans* and bacterial microbiota interactions in the cecum during recolonization following broad-spectrum antibiotic therapy. Infect Immun.

[37] Tan CT, Xu X, Qiao Y, Wang Y (2021). A peptidoglycan storm caused by β-lactam antibiotic's action on host microbiota drives *Candida*
*albicans* infection. Nat Commun.

[38] Pan S, Huang X, Wang Y, Li L, Zhao C, Yao Z (2018). Efficacy of intravenous plus intrathecal/intracerebral ventricle injection of polymyxin B for post-neurosurgical intracranial infections due to MDR/XDR *Acinectobacter*
*baumannii*: a retrospective cohort study. Antimicrob Resist Infect Control.

[39] Rodrigues ME, Gomes F, Rodrigues CF (2019). Candida spp./bacteria mixed biofilms. J Fungi (Basel, Switzerland)..

[40] Carlson E (1982). Synergistic effect of *Candida*
*albicans* and *Staphylococcus*
*aureus* on mouse mortality. Infect Immun.

[41] Effah CY, Sun T, Liu S, Wu Y (2020). *Klebsiella*
*pneumoniae*: an increasing threat to public health. Ann Clin Microbiol Antimicrob.

[42] Dogan O, Yesilkaya A, Menekse S, Guler O, Karakoc C, Cinar G (2020). The effect of initial antifungal therapy on fatality among the patients with blood stream infections with different Candida species and resistance to antifungal agents: a multicenter observational study of the Turkish Fungal Infections Study Group. Int J Antimicrob Agents..

