# Supplementary figures and images for: Incidence, clinical characteristics, risk factors and outcomes of patients with mixed Candida/bacterial bloodstream infections: a retrospective study
Source: Ann Clin Microbiol Antimicrob. 2022 Nov 1;21:45. doi: 10.1186/s12941-022-00538-y (PMC9628097; doi:10.1186/s12941-022-00538-y)

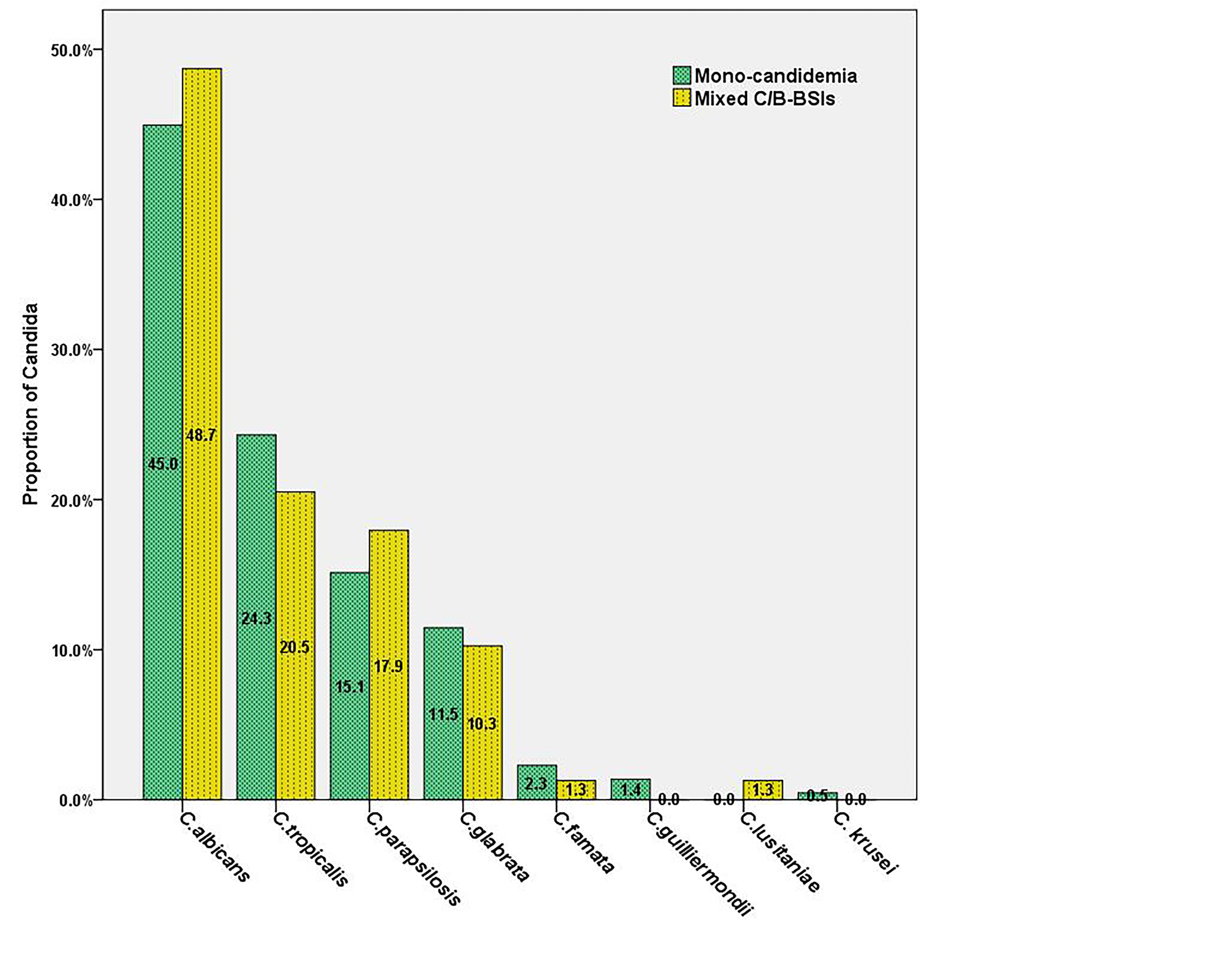

Supplement: Supplementary file 1 — Additional file 1: Figure S1. The distribution comparison of Candida species isolated from mixed C/B-BSIs and mono-candidemia. Mono-candidemia, monomicrobial candidemia; Mixed C/B-BSIs, mixed Candida/bacterial bloodstream infections. [file 12941_2022_538_MOESM1_ESM.jpg]
