# Supplementary material for: Incidence, clinical characteristics, risk factors and outcomes of patients with mixed Candida/bacterial bloodstream infections: a retrospective study
Source: Ann Clin Microbiol Antimicrob. 2022 Nov 1;21:45. doi: 10.1186/s12941-022-00538-y (PMC9628097; doi:10.1186/s12941-022-00538-y)
Supplement: Supplementary file 2 — Additional file 2: Table S1. Comparison of vitro antifungal susceptibility of candida between mono-candidemia and mixed C/B-BSIs. [file 12941_2022_538_MOESM2_ESM.docx]

**Table S1**. Comparison of vitro antifungal susceptibility of candida between mono-candidemia and mixed C/B-BSIs.

| *Candida* species and antifungal | Mono-candidemia  (n=218) | | | | Mixed C/B-BSIs  (n=78) | | | | |
| --- | --- | --- | --- | --- | --- | --- | --- | --- | --- |
|  | Number of strains | S | I | R | Number of strains | S | I | R | *P* value  (S) |
| ***C.albicans*** (n=136) | | | | | | | | | |
| FLC(n=123) ^a^ | 91(74.0) | 88(96.7) | 3(3.1) | 0 | 32(26.0) | 31(96.9) | 1(2.6) | 0 | >0.99 |
| CLO(n=66) ^a^ | 46(69.7) | 45(97.8) | 0 | 1(1.2) | 20(30.3) | 20(100.0) | 0 | 0 | 0.48 |
| KET(n=58) ^a^ | 37(63.8) | 15(40.5) | 13(35.1) | 9(24.3) | 21(36.2) | 9(42.9) | 8(38.1) | 4(19.0) | 0.86 |
| ITR(n=130) ^a^ | 94(72.3) | 90(95.7) | 2(2.1) | 2(2.1) | 36(27.7) | 35(97.2) | 1(2.8) | 0 | >0.99 |
| AMB (n=130) ^a^ | 95(73.1) | 95(100) | 0 | 0 | 35(26.9) | 35(100.0) | 0 | 0 | >0.99 |
| 5-FLU(n=61) ^a^ | 46(75.4) | 45(97.8) | 0 | 1(2.2%) | 15(24.6) | 15(100) | 0 | 0 | >0.99 |
| VOR(n=118) ^a^ | 86(72.9) | 86(100.0) | 0 | 0 | 32(27.1) | 32(100.0) | 0 | 0 | >0.99 |
| ***C.tropicalis*** (n=69) | | | | | | | | | |
| FLC(n=67) ^a^ | 52(77.6) | 24(46.2) | 3(5.8) | 25(48.1) | 15(22.4) | 8(53.3) | 1(6.7) | 6(40.0) | 0.62 |
| CLO(n=34) ^a^ | 24(70.6) | 8(33.3) | 13(54.2) | 3(12.5) | 10(29.4) | 4(40.0) | 4(40.0) | 2(20.0) | >0.99 |
| KET(n=34) ^a^ | 23(67.6) | 5(21.7) | 10(43.5) | 8(34.8) | 11(32.4) | 4(36.4) | 3(27.3) | 4(36.4) | 0.62 |
| ITR(n=66) ^a^ | 51(77.3) | 20(39.2) | 8(15.7) | 23(45.1) | 15(22.7) | 10(66.7) | 0 | 5(33.3) | 0.06 |
| AMB (n=69) ^a^ | 53(76.8) | 52(98.1) | 0 | 1(1.9) | 16(23.2) | 16(100.0) | 0 | 0 | >0.99 |
| NYS(n=33) ^a^ | 23(69.7) | 22(95.7) | 1(4.3) | 0 | 10(30.3) | 10(100.0) | 0 | 0 | >0.99 |
| 5-FLU(n=33) ^a^ | 28(84.8) | 28(100.0) | 0 | 0 | 5(15.2) | 5(100.0) | 0 | 0 | >0.99 |
| VOR(n=58) ^a^ | 46(79.3) | 21(45.7) | 0 | 25(54.3) | 12(20.7) | 6(50.0) | 0 | 6(50.0) | 0.79 |
| ***C.parapsilosis*** (n=47) | | | | | | | | | |
| FLC(n=43) ^a^ | 30(69.8) | 29(96.7) | 0 | 1(3.3) | 13(30.2) | 11(84.6) | 2(15.4) | 0 | 0.21 |
| CLO(n=22) ^a^ | 17(77.3) | 15(88.2) | 1(5.9) | 1(5.9) | 5(22.7) | 5(100.0) | 0 | 0 | >0.99 |
| KET(n=22) ^a^ | 16(72.7) | 11(68.8) | 2(12.5) | 3(18.7) | 6(27.3) | 1(16.7) | 4(66.6) | 1(16.7) | 0.09 |
| ITR(n=40) ^a^ | 27(67.5) | 25(92.6) | 2(7.4) | 0 | 13(32.5) | 12(92.3) | 1(7.7) | 0 | >0.99 |
| AMB (n=46) ^a^ | 32(69.6) | 32(100.0) | 0 | 0 | 14(30.4) | 14(100.0) | 0 | 0 | >0.99 |
| NYS(n=22) ^a^ | 16(72.7) | 16(100.0) | 0 | 0 | 6(27.3) | 6(100.0) | 0 | 0 | >0.99 |
| 5-FLU(n=21) ^a^ | 13(61.9) | 13(100.0) | 0 | 0 | 8(38.1) | 8(100.0) | 0 | 0 | >0.99 |
| VOR(n=40) ^a^ | 27(67.5) | 26(96.3) | 0 | 1(3.7) | 13(32.5) | 13(100.0) | 0 | 0 | >0.99 |
| ***C.glabrata*** (n=33) | | | | | | | | | |
| FLC(n=31) ^a^ | 23(74.2) | 17(73.9) | 4(17.4) | 2(8.7) | 8(25.8) | 8(100.0) | 0 | 0 | 0.30 |
| CLO(n=19) ^a^ | 17(85.0) | 12(70.6) | 3(17.6) | 2(11.8) | 3(15.0) | 2(66.7) | 0 | 0 | >0.99 |
| KET(n=19) ^a^ | 18(94.7) | 7(38.9) | 4(22.2) | 7(38.9) | 1(5.3) | 1(100.0) | 0 | 0 | 0.42 |
| ITR(n=27) ^a^ | 21(77.8) | 12(57.1) | 5(23.8) | 4(19.0) | 6(22.2) | 3(50.0) | 2(33.3) | 1(16.7) | >0.99 |
| AMB (n=33) ^a^ | 25(75.8) | 25(100.0) | 0 | 0 | 8(24.2) | 8(100.0) | 0 | 0 | >0.99 |
| NYS(n=22) ^a^ | 20(90.9) | 20(100.0) | 0 | 0 | 2(9.1) | 2(100.0) | 0 | 0 | >0.99 |
| 5-FLU(n=11) ^a^ | 5(45.5) | 5(100.0) | 0 | 0 | 6(54.5) | 6(100.0) | 0 | 0 | >0.99 |
| VOR(n=28) ^a^ | 20(71.4) | 19(95.0) | 0 | 1(5.0) | 8(28.6) | 7(87.5) | 0 | 1(12.5) | 0.50 |
| ***C. famata*** (n=6) | | | | | | | | | |
| FLC(n=5) ^a^ | 4(80.0) | 4(100.0) | 0 | 0 | 1(20.0) | 1(100.0) | 0 | 0 | >0.99 |
| CLO(n=5) ^a^ | 4(80.0) | 4(100.0) | 0 | 0 | 1(20.0) | 1(100.0) | 0 | 0 | >0.99 |
| KET(n=4) ^a^ | 3(75.0) | 2(66.7) | 1(33.3) | 0 | 1(25.0) | 1(100.0) | 0 | 0 | >0.99 |
| ITR(n=5) ^a^ | 4(80.0) | 4(100.0) | 0 | 0 | 1(20.0) | 1(100.0) | 0 | 0 | >0.99 |
| AMB (n=6) ^a^ | 5(83.3) | 5(100.0) | 0 | 0 | 1(16.7) | 1(100.0) | 0 | 0 | >0.99 |
| NYS(n=4) ^a^ | 3(75.0) | 3(100.0) | 0 | 0 | 1(25.0) | 1(100.0) | 0 | 0 | >0.99 |
| 5-FLU(n=2) ^a^ | 2(100.0) | 2(100.0) | 0 | 0 | 0 | 0 | 0 | 0 |  |
| VOR(n=3) ^a^ | 2(66.7) | 2(100.0) | 0 | 0 | 1(33.3) | 1(100.0) | 0 | 0 | >0.99 |
| ***C. guilliermondii***  (n= 3) | | | | | | | | | |
| FLC(n=3) ^a^ | 3(100.0) | 1(33.3) | 1(33.3) | 1(33.3) | 0 | 0 | 0 | 0 |  |
| CLO(n=2) ^a^ | 2(100.0) | 2(100.0) | 0 | 0 | 0 | 0 | 0 | 0 |  |
| KET(n=3) ^a^ | 3(100.0) | 3(100.0) | 0 | 0 | 0 | 0 | 0 | 0 |  |
| ITR(n=3) ^a^ | 3(100.0) | 3(100.0) | 0 | 0 | 0 | 0 | 0 | 0 |  |
| AMB (n=3) ^a^ | 3(100.0) | 3(100.0) | 0 | 0 | 0 | 0 | 0 | 0 |  |
| NYS(n=3) ^a^ | 3(100.0) | 3(100.0) | 0 | 0 | 0 | 0 | 0 | 0 |  |
| VOR(n=3) ^a^ | 3(100.0) | 3(100.0) | 0 | 0 | 0 | 0 | 0 | 0 |  |
| ***C. krusei*** (n=1) | | | | | | | | | |
| FLC(n=1) ^a^ | 1(100.0) | 0 | 0 | 1(100.0) | 0 | 0 | 0 | 0 |  |
| CLO(n=1) ^a^ | 1(100.0) | 1(100.0) | 0 | 0 | 0 | 0 | 0 | 0 |  |
| KET(n=1) ^a^ | 1(100.0) | 0 | 0 | 1(100.0) | 0 | 0 | 0 | 0 |  |
| ITR(n=1) ^a^ | 1(100.0) | 0 | 1(100.0) | 0 | 0 | 0 | 0 | 0 |  |
| AMB (n=1) ^a^ | 1(100.0) | 1(100.0) | 0 | 0 | 0 | 0 | 0 | 0 |  |
| NYS(n=1) ^a^ | 1(100.0) | 1(100.0) | 0 | 0 | 0 | 0 | 0 | 0 |  |
| VOR(n=1) ^a^ | 1(100.0) | 1(100.0) | 0 | 0 | 0 | 0 | 0 | 0 |  |
| ***C. lusitaniae*** (n=1) | | | | | | | | | |
| FLC(n=1) ^a^ | 0 | 0 | 0 | 0 | 1(100.0) | 1(100.0) | 0 | 0 |  |
| ITR(n=1) ^a^ | 0 | 0 | 0 | 0 | 1(100.0) | 1(100.0) | 0 | 0 |  |
| AMB (n=1) ^a^ | 0 | 0 | 0 | 0 | 1(100.0) | 1(100.0) | 0 | 0 |  |
| 5-FLU(n=1) ^a^ | 0 | 0 | 0 | 0 | 1(100.0) | 1(100.0) | 0 | 0 |  |
| VOR(n=1) ^a^ | 0 | 0 | 0 | 0 | 1(100.0) | 1(100.0) | 0 | 0 |  |

**Notes**: S, sensitive; I, intermediary; R, resistant. FLC, fluconazole; CLO, clotrimazole; KET: ketoconazole; ITR: itraconazole; AMB, amphotericin B; NYS: nystatin; 5-FLU, 5-fluorocytosine; VOR, voriconazole;

^a^ Not all agents listed tested in all isolates, the figures in parentheses were the total numbers of *Candida* used for antifungal susceptibility testing in both groups.
